# Relationship between MRI derived right ventricular mass and left ventricular involvement in patients with anderson-fabry disease

**DOI:** 10.1186/1532-429X-17-S1-P275

**Published:** 2015-02-03

**Authors:** Qin Li, Ming-Yen Ng, Anna Calleja, Djeven P Deva, Andrew Crean, Christiane Gruner, Robert M Iwanochko, Paaladinesh Thavendiranathan

**Affiliations:** Radiology, University of Toronto, Toronto, ON Canada; Radiology, The University of Hong Kong, Hong Kong, Hong Kong; Cardiology, University of Toronto, Toronto, ON Canada; Radiology, St. Michael’s Hospital, Toronto, ON Canada

## Background

Anderson-Fabry's Disease is an X-linked lysosomal storage disorder in which there is an accumulation of globotriaosylceramide in both atria and ventricles as well as valvular tissue. We sought to investigate if there was a correlation on cardiac MRI between the degree of left ventricular (LV) involvement (ie. LV mass or late gadolinium enhancement) and right ventricular mass.

## Methods

**Figure 1:**
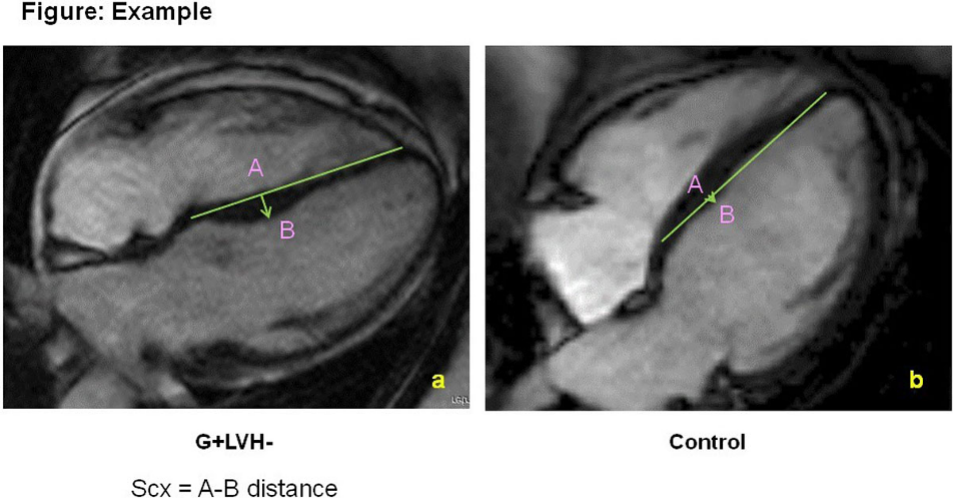
Assessment of RV volume, function, and mass using short axis SSFP cine images (CMR42). The RV septal band was included in the assessment of the RV mass.

The study was approved by the research ethics board. Patients with Anderson-Fabry's disease were identified through the Metabolic Genetic Disease Clinic between 1^st^ January 2000 and 31^st^ December 2013. We included only patients with genotyping and leucocyte alpha galactosidase A activity test confirming a diagnosis of Anderson-Fabry's disease. Clinical data obtained included age and body mass index. Cardiac MRI was analysed using CMR 42 (Circle Cardiovascular Imaging) to measure LV and right ventricular (RV) volumes, ejection fraction and mass using short axis steady state free precession (SSFP) cine images (see figure 1). Presence of late gadolinium enhancement (LGE) and degree of LGE was calculated using a threshold of 6 SD above a remote region. Right ventricular hypertrophy (RVH) was defined as >28g/m^2^ for males and >27g/m^2^ for women. This threshold was based on a paper by Hudsmith et al (2005 Journal of Cardiovascular Magnetic Resonance).

Comparisons between RV mass, LV mass and LGE were performed using Pearson's correlation and unpaired student t-test.

## Results

43 patients (22 males) with a mean age of 45 ± 14yrs were included. There was good correlation between LV and RVEF (r=0.59, p<0.01), and LV and RV mass index (r=0.67, p<0.01). Total LV scar was higher in patients with right ventricular hypertrophy (RVH) (12.48g ± 24g) than in those without RVH (1.44g ± 2.29g) (p=0.0047). There was also a statistically significant difference between the LV mass of patients with increased RV mass (117.03g/m^2^ +/- 69g/m^2^) compared to patients with normal RV mass (71g/m^2^ +/- 28.7g/m^2^) (see table 1).

**Table 1:** Comparison of LV mass and LGE quantification in Anderson-Fabry's patients with increased and normal RV mass with p-values from unpaired t-test.

	Increased RV mass	Normal RV mass	p-value
LV mass (g/m2)	117.03 ±69	71 ±28.7	0.0146
LGE 6SD (g)	12.48 ±24	1.44 ±2.29	0.0047

## Conclusions

Patients with Anderson-Fabry's disease with increased LV mass and LGE had a positive correlation with increased RV mass. RV mass changes appear to occur in more advanced myocardial involvement with Anderson-Fabry's disease.

## Funding

None.

