# Erratum: Relationship between MRI derived right ventricular mass and left ventricular involvement in patients with anderson-fabry disease

**DOI:** 10.1186/s12968-015-0135-z

**Published:** 2015-05-29

**Authors:** Ming-Yen Ng, Qin Li, Anna Calleja, Djeven P Deva, Andrew M Crean, Christiane Gruner, Robert M Iwanochko, Paaladinesh Thavendiranathan

**Affiliations:** Radiology, University of Toronto, Toronto, ON Canada; Radiology, The University of Hong Kong, Hong Kong, Hong Kong; Cardiology, University of Toronto, Toronto, ON Canada; Radiology, St. Michael’s Hospital, Toronto, ON Canada

Following publication of this abstract [1] it was noted by the authors that an incorrect version of Figure 1 had been inadvertently uploaded with the manuscript. The correct version Figure 1 is available below.

**Figure 1:**
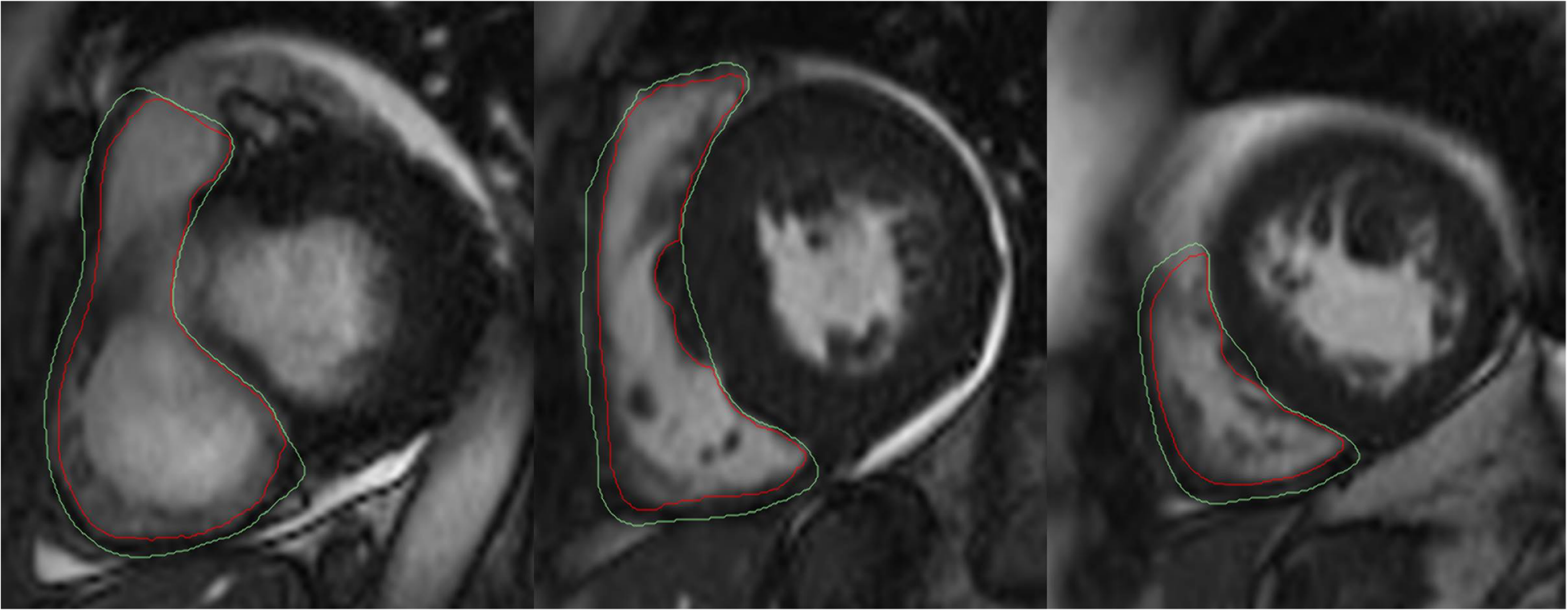
Assessment of RV volume, function, and mass using short axis SSFP cine images (CMR42). The RV septal band was included in the assessment of the RV mass.

In addition it was found that the author list was incorrectly ordered, this has been corrected in the author list above.
